# An Unusual Case of Anterior Mediastinal Cystic Echinococcosis Successfully Resolved with Multidisciplinary Approach

**DOI:** 10.3390/pathogens14101016

**Published:** 2025-10-07

**Authors:** Katarzyna Rodak, Magdalena Mnichowska-Polanowska, Arkadiusz Waloryszak, Konrad Ptaszyński, Janusz Wójcik, Małgorzata Edyta Wojtyś

**Affiliations:** 1 Department of Pathology, Regional Hospital SPWSZ in Szczecin, 71-455 Szczecin, Poland; 2 Department of Microbiology Immunology and Laboratory Medicine, Pomeranian Medical University in Szczecin, Powstańców Wielkopolskich 72, 70-111 Szczecin, Poland; 3 Department of Thoracic Surgery and Transplantation, Pomeranian Medical University in Szczecin, Alfreda Sokołowskiego 11, 70-891 Szczecin, Poland

**Keywords:** *Echinococcus*, cystic echinococcosis, diagnosis, histopathology, anterior mediastinum, serology

## Abstract

Human echinococcosis is a zoonotic disease caused by accidental ingestion of tapeworm eggs of the genus *Echinococcus*, shed in the feces of animal definitive host. In the human duodenum, these eggs release oncospheres, which penetrate the intestinal wall and via the bloodstream reach the liver—the most common site for development of cysts. However, it is important to remember that any other organ can be affected via the bloodstream, due to larvae size. In Europe, the most diagnostically relevant species are *Echinococcus granulosus*, with a median incidence of 0.6 cases per 100,000 inhabitants, and *Echinococcus multilocularis*, with 0.1 cases per 100,000 inhabitants. This article aims to describe an exceptionally unusual location of human cystic echinococcosis in the anterior mediastinum. We describe the role of multidisciplinary diagnostics in establishing the definitive diagnosis. The pathomorphological examination, radiological imaging and serological testing for diagnosing cystic echinococcosis are hereby described. It is particularly important to avoid reporting unspecified *Echinococcus* (NOS) if possible, as the management and treatment of patients with echinococcosis varies depending on the species.

## 1. Introduction

Human echinococcosis is a zoonotic infection caused by cestode species of the genus *Echinococcus* [1]. *Echinococcus granulosus* is present worldwide and relatively frequent in regions with poor veterinary surveillance. In Europe, including Poland, the most relevant species diagnostically are *Echinococcus granulosus* and *Echinococcus multilocularis*, which most commonly cause cystic echinococcosis (CE) and alveolar echinococcosis (AE), respectively [2]. In Poland, according to data from the Sanitary and Epidemiological Station reports, the mean annual number of echinococcosis cases in the years 2010–2023 was 45, with an incidence rate of 0.12 per 100,000 population, while the most recent data from 2023 reported 65 cases [3]. According to the European Centre for Disease Prevention and Control (ECDC), in 2022 the notification rate for *Echinococcus* spp. was 0.19 cases per 100,000 population. Sixteen countries reported 299 confirmed cases of cystic echinococcosis caused by *E. granulosus* and thirteen countries reported 185 cases of alveolar echinococcosis caused by *E. multilocularis* [4]. Median incidence per 100,000 inhabitants was calculated at 0.6 (95% UI 0.4–5) for CE and 0.1 (95% UI 0.01–0.2) for AE. [2] Discriminating between AE and CE is important because they require different approaches to patient management [5]. *E. granulosus* still requires clinical awareness and diagnostic expertise to diagnose the infection earlier.

Humans do not participate in the natural cycle of *E. granulosus* and are considered an accidental or aberrant intermediate host. Human infection (CE or hydatid disease) typically occurs after the accidental ingestion of parasite eggs, which are excreted into the environment (i.e., soil, food, water) via the feces of domestic and wild canids or may be present on the fur of these animals [1,6,7]. The eggs of this cestode contain infective oncospheres. After accidental ingestion by a human, these oncospheres penetrate the intestine and then migrate via the bloodstream to internal vital organs, commonly the liver or lungs [1,7,8]. After organ infection, the larval stage, known as the metacestode, forms. The growth and expansion of metacestodes within organs cause the formation of pathological fluid-filled lesions (hydatid cysts) with highly antigenic substances [1,5]. The World Health Organization Informal Working Group on Echinococcosis (WHO-IWGE) has classified CE cysts into five types and three groups [9]. CE cysts in the infected organ can displace to another organ and pass through different stages, from unilocular fluid-filled CE1 to more complex active stages (CE2, CE3a, CE3b) and solid inactive stages (CE4, CE5) [5,10]. Clinical manifestations differ according to the location, size, and number of cysts.

Most *E. granulosus* infections are asymptomatic for a long time, and the incubation period is variable. Clinical symptoms will appear after the infection reaches a certain size because of the pressure exerted on nearby tissues [11,12]. Hydatid cysts contain brood capsules with numerous viable protoscolices suspended in hydatid fluid. Their spontaneous or surgical rupture can lead to secondary echinococcosis (relapses) and the induction of a systemic immune response [11,13]. In 70% of cases, *E. granulosus* is typically located in the liver, but it can also be found in the lungs and unique intrathoracic extrapulmonary locations, including the mediastinum [14,15]. Mediastinal hydatid cysts are extremely uncommon, accounting for less than 0.1% of all reported cases [16], while overall 0.5–2.6% of hydatid cysts are found in the mediastinum [15]. Primary mediastinal hydatid cysts are most commonly located in the posterior, anterior, and middle mediastinum [16,17].

## 2. Aim of the Study

Here, we describe a well-documented, extremely rare case of cystic echinococcosis in the anterior mediastinum that was initially misdiagnosed as a parasitic cyst. Due to the lack of clinical and radiological suspicion, as well as the absence of prior patient history and anthelmintic treatment, the pathological examination played a significant role in the diagnosis. To the best of our knowledge, this is the first reported case of cystic echinococcosis in a patient from Poland, located in the anterior mediastinum and developed over 50 years following the primary infection, successfully diagnosed and resolved with a multidisciplinary approach, as described in this paper.

## 3. Case Presentation

**Figure 1:**
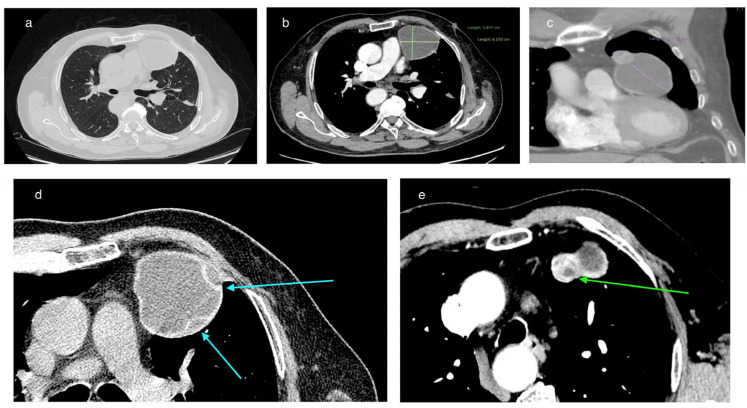
CT imaging of a cyst in the left anterior mediastinum. (**a**) Axial chest CT images in the lung window without contrast enhancement. (**b**) Contrast-enhanced axial chest CT images with the long axis measurement. (**c**) Contrast-enhanced coronal chest CT images with delineation of its long axis measurement. (**d**) Focal detachment of the cyst’s internal wall – indicated by the arrow. (**e**) Contrast-enhanced nodular mass adjacent to the fluid-filled cyst – indicated by the arrow.

History of the Patient and radiological examination:

In February 2025, a 69-year-old patient was admitted to the Department of Thoracic Surgery and Transplantology at Pomeranian Medical University in Szczecin for surgical treatment of a mediastinal lesion. Upon admission, the patient reported intermittent chest pain. Chest computed tomography (CT) performed 25 November 2024 had revealed a well-defined, cystic, hypodense lesion in the left anterior mediastinum with an attenuation of 3–7 Hounsfield Units (HU) and measuring (in vivo) approximately 58 mm × 62 mm (axial plane) × 77 mm (long axis) (Figure 1). The cyst walls measured up to 4 mm and had a segmental detached membrane forming thin, flattened pseudo-vesicles (Figure 1). At the upper pole of the cyst, a heterogeneous dense (50 HU) nodular mass exhibited mild contrast enhancement and measured approximately 25 mm in diameter (Figure 1). These findings were equivocal and lacked distinctive features to establish the diagnosis of a pericardial cyst, thymic cyst, hamartoma, or cystic proliferation (lymphoma/meta) of the pericardium or lung. Based on the patient’s history of previous left-sided chest CE and left posterolateral thoracotomy, the differential diagnosis of the mediastinal lesion detected >50 years later should include hydatid cyst.

**Figure 2:**
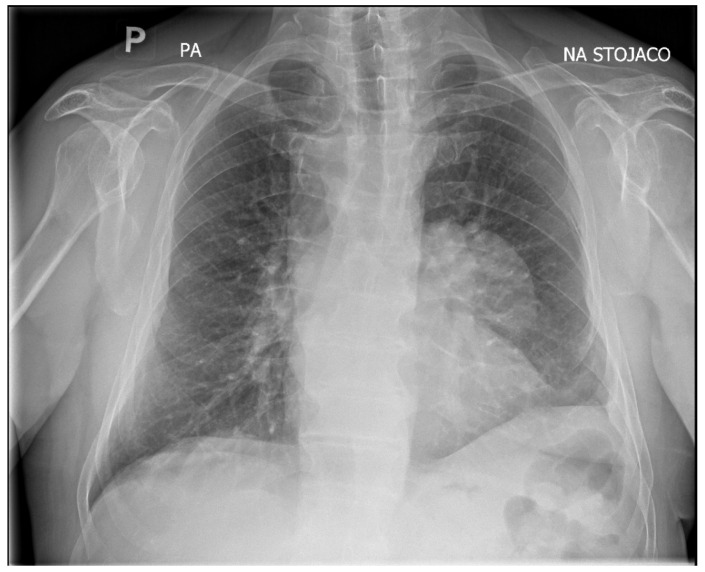
Chest X-ray showing well-defined consolidation in the left pulmonary hilum. PA, posteroanterior position.

Upon admission, the patient underwent chest X-ray, which demonstrated well-defined consolidation in the left pulmonary hilum (Figure 2). An echocardiogram revealed that the lesion was not communicating with the heart muscle. However, it was adjacent to the pericardium.

In 1966, the patient underwent a left posterolateral thoracotomy due to CE in the left lung. His medical history revealed that he had not received any subsequent antiparasitic treatment and had not been followed up. Since that surgery, he had not reported any complaints or symptoms suggestive of an active *Echinococcus* infection. The patient’s comorbidities included hypertension, which was controlled by pharmacological treatment, and chronic autoimmune thyroiditis (Hashimoto’s thyroiditis).

Surgical treatment:

Due to the absence of medical contraindications, the patient was scheduled for elective surgical removal of the lesion. A thoracotomy was performed, with excision of the old scar and removal of the left fifth rib. The pleural cavity was obliterated, and a sharp detachment was performed within the upper lobe without interfering with the lower lobe. A thick-walled cyst measuring approximately 80 × 70 mm was visualized in the anterior mediastinum. This estimated visual dimension during thoracotomy, which is an approximate assessment, may be disturbed by the position of the cyst in the intraoperative field, hence the difference in relation to the dimensions from CT. This was the traditional surgical technique for mediastinal cystic lesions. The tissues were gently dissected and the lesion was enucleated using blunt dissection without rupturing the cyst wall. The operation had to be performed carefully so as not to rupture the cyst wall and cause contamination. After thorough examination, no focal lesions were found in the left lung.

**Figure 3:**
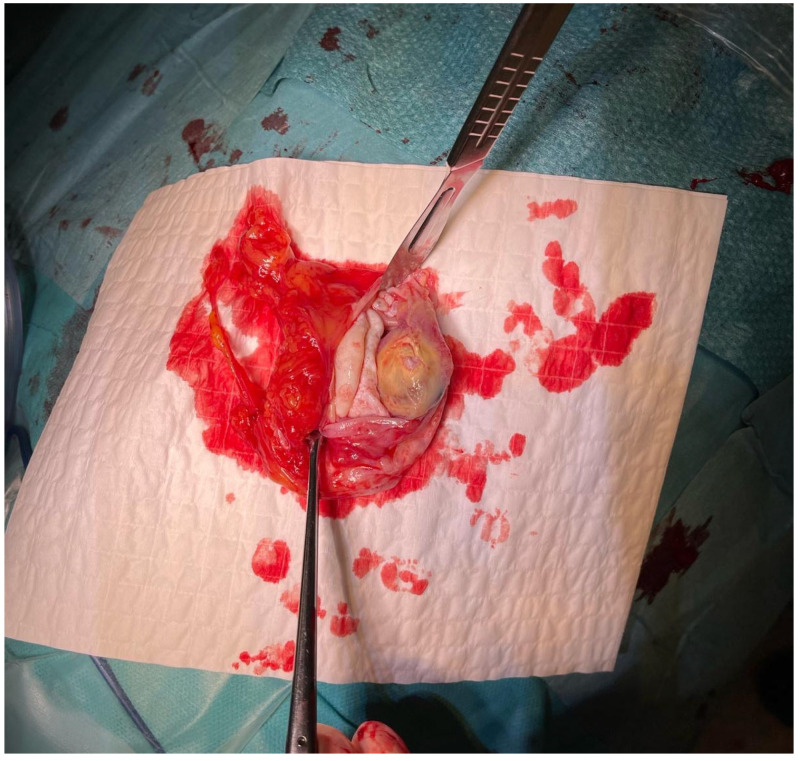
Intraoperative image of the *Echinoccocus* spp. cyst removed from the patient’s anterior mediastinum.

**Figure 4:**
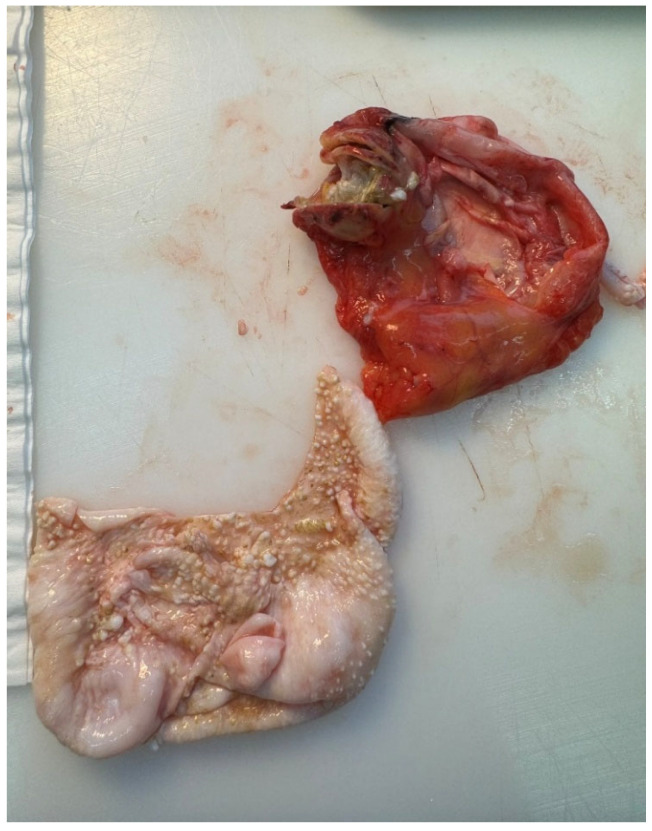
Dissection of the *Echinococcus* spp. cyst.

**Figure 5:**
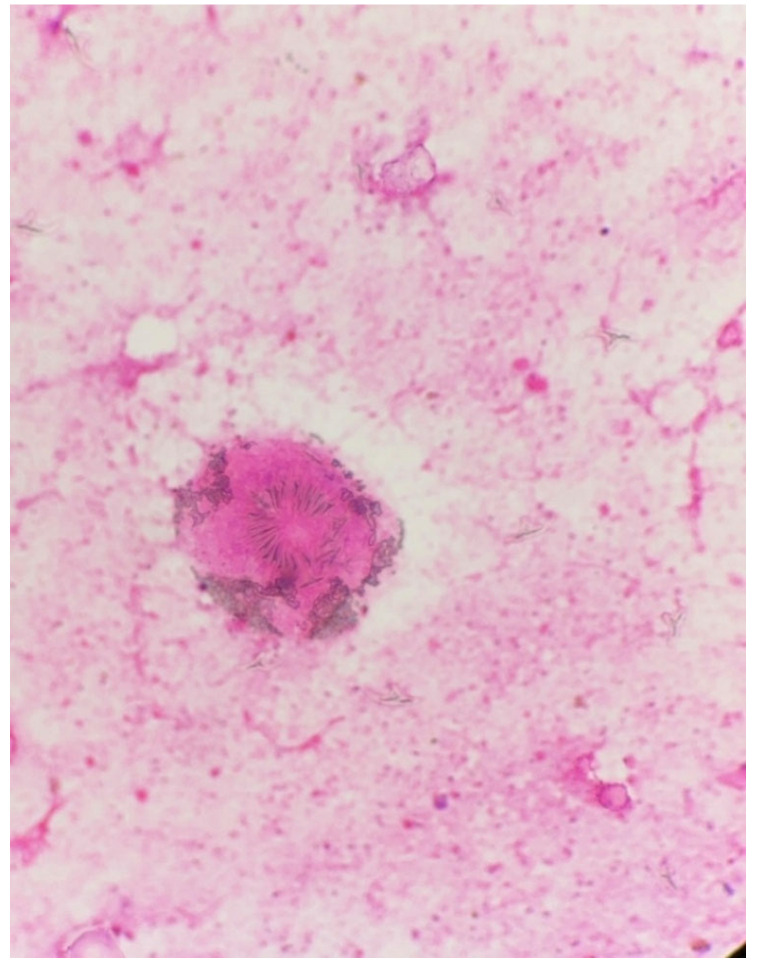
Intraoperative light microscopy images showing the viable protoscolex of *Echinococcus* spp. stained with H&E (40×).

Pathology and histopathology examination:

The material was submitted to intraoperative examination (Figure 3). The cyst measured (ex vivo) 95 mm × 61 mm × 22 mm (Figure 4). The dimensions of the postoperative specimen are the ex vivo dimensions after removal and dissection of the cyst. The cyst flattened after fluid removal, explaining the difference in dimensions compared to the CT scan, which showed the cyst in situ, filled with fluid. Upon dissection, it had an uneven whitish internal surface with nodular thickening. Biopsies were collected for histological and cytological examination. The cyst fluid had not previously been aspirated for independent analysis. Cytological examination involved making a smear from the inner surface of the cyst wall, but only biopsies from the cyst wall, not from the nodular portion, were collected for histological examination. The cyst wall showed signs of necrosis and the presence of histiocytes and lymphoid cells. Cytological examination revealed structures consistent with protoscolex (Figure 5), suggesting *Echinococcus* spp.

**Figure 6:**
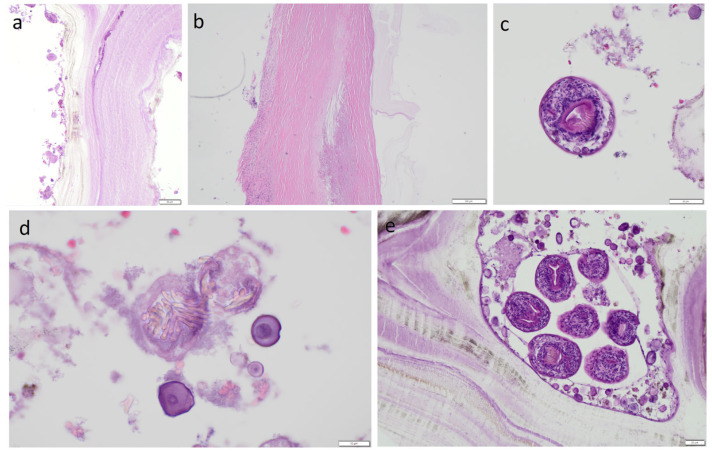
Pathomorphological examination. (**a**) Germinal layer and lamellar layer. H&E staining, 10×. (**b**) Pericyst. H&E staining, 4×. (**c**) Protoscolex. H&E staining, 40×. (**d**) Aggregation of hooklets. H&E staining, 40×. (**e**) Brood capsule containing protoscolices. H&E staining, 20×. Scale bars: (**a**) 50 μm, (**b**) 200 μm, (**c**) 20 μm, (**d**) 10 μm, (**e**) 20 μm.

The material was fixed in formalin to prepare hematoxylin and eosin (H&E)-stained slides. Dissection of the cyst caused the liquid filling it to spill over its surface and may have contained numerous protoscolices, the so-called hydatide sand. We took samples from both the nodular lesion and the cyst wall. In both of these locations, protoscolices were visible under the microscope after H&E staining, but there were more of them in the sections from the nodular lesion. However, whether the protoscolices were transferred from one location to another due to the dissection of the cyst cannot be determined. Because only a few protoscolex were found in the nodular lesion, it appears to be the result of contamination of this area. However, this is hypothetical. Therefore, differentiating between viable daughter cysts and reactive secondarily seeded tissue in the nodular lesion is problematic. The slides revealed a cyst wall composed of a germinal (inner) layer and an outer layer with a 0.40-mm-thick concentrically arranged acellular layer of lamellae (Figure 6). The outer layer was surrounded by a pericyst, a fibrous capsule resulting from the host response (i.e., not part of the parasitic tissues). The pericyst was up to 2.2-mm-thick and contained clusters of histiocytes, macrophages, and inflammatory cells [Figure 6] The examined slides also revealed numerous protoscolices with hooklet rings and suckers (Figure 6), free hooklets (Figure 6), and a brood capsule containing protoscolices, which are produced by the germinal membrane (Figure 6). This image was consistent with the diagnosis of a *E. granulosus* cyst.

Serological testing:

A blood sample (approximately 7.5 mL) was collected from the patient after the surgical procedure and the serum was separated and immediately tested using a two-stage serological approach. The serum was not frozen. This case of mediastinal echinococcosis was diagnosed serologically with a sensitivity of 96% as declared by the manufacturer of the *E. granulosus* enzyme-linked immunosorbent assay (ELISA) screening assay sensitized with *E. granulosus* hydatid fluid antigen (Bordier Affinity Products, no. 9350). Simultaneously, the sample was retested with Em2-Em18 antigens specific for *E. multilocularis*-specific (Bordier Affinity Products, no. 9300) to identify the *Echinococcus species*.

The absorbance at 405 nm was read using an ELISA microplate reader. The test results were expressed as an index (absorbance of the sample divided by absorbance cut-off for serum) and validated with criteria provided by the manufacturer (Bordier). A result was considered positive and clinically significant when the index was >1.0; the higher the index value, the more IgG antibodies detected against *E. granulosus* and/or *E. multilocularis*.

For confirmation of *Echinococcus*-specific IgG antibodies and identification of the parasite species, we used the anti-*Echinococcus* blot assay EUROLINE-WB (IgG) (ELB, Euroimmun). For differential diagnostics, this kit included separated native antigen extract of *Echinococcus*, recombinant *E. granulosus*-specific antigens (EgAgB), and recombinant species-specific *E. multilocularis* antigens (Em18 and Em95). With this assay, the differentiation rate between *E. granulosus* and *E. multilocularis* was 81% according to the manufacturer. An experienced laboratory diagnostician (microbiologist and parasitologist) interpreted the results and read the ELB strips.

**Figure 7:**
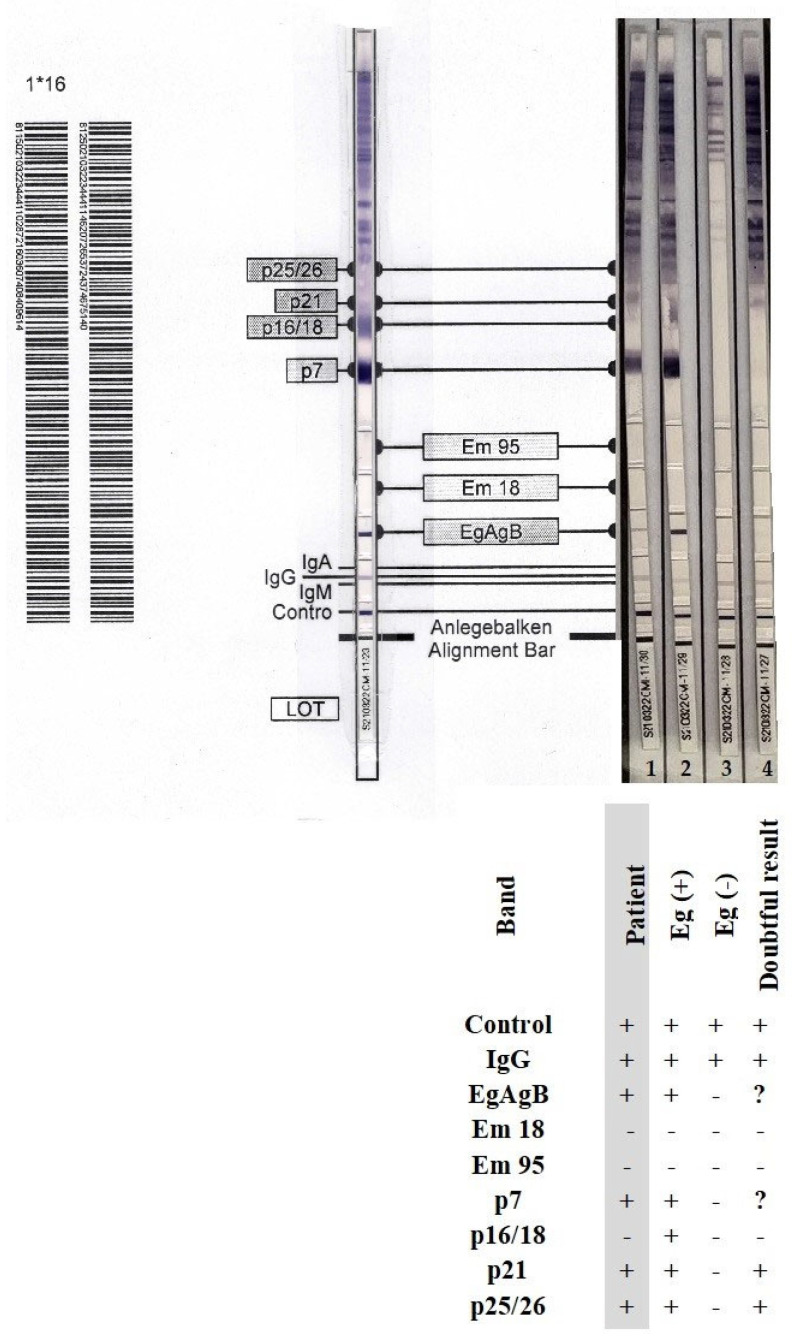
Serological confirmation of mediastinal echinococcosis using anti-*Echinococcus* EUROLINE-WB (IgG) and differential analysis between *E. granulosus* and *E. multilocularis*. Strip 1 (left, gray): Positive result for *E. granulosus* based on the genus-specific band EgAgB and two immunoreactive bands, p7 (7 kDa) and p21 (21 kDa). No bands specific for *E. multilocularis* were detected. Strip 2: Positive control sample for *E. granulosus*. Strip 3: Negative control sample for *E. granulosus*. Strip 4: No discrimination between *E. granulosus* and *E. multilocularis*. “?” – Inconclusive result; the signal is very weak, diffuse, or hard to interpret.

**Figure 8:**
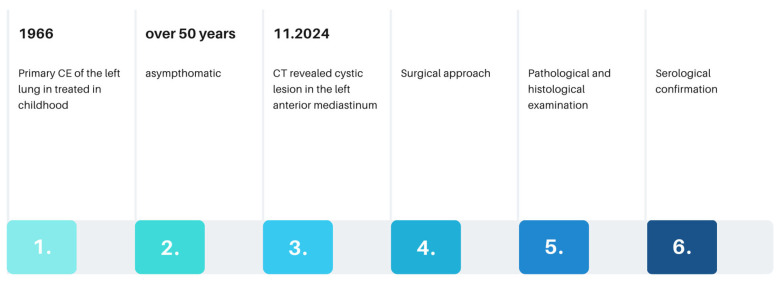
Patient’s history timeline.

The concentration of IgG antibodies against *E. granulosus* antigen was considered clinically significant, with an index value of 6.12. In contrast, the concentration of IgG antibodies against *E. multilocularis* Em2-Em18 antigens was clinically non-significant with an index value of 0.89. Using the ELB assay, we could differentiate between *E. granulosus* and *E. multilocularis* based on the presence of the following immunoreactive bands: EgAgB, p7 (7 kDa), p21 (21 kDa), and p25/26 (24–26 kDa). The patient’s serological result was indicative of *E. granulosus* infection, as the genus-specific EgAgB band and two additional diagnostic bands, p7 and p21 (corresponding to category 3 and 4, respectively), were identified in accordance with the differential interpretation criteria established by the assay manufacturer; no bands specific for *E. multilocularis* Em18 and/or Em95 were detected (Figure 7). The detection of parasite-specific antibodies in the patient’s serum corroborated the intraoperative observations of well-delineated cysts and findings from the histopathological examination. Figure 8 illustrates the timeline of the patient’s medical history.

## 4. Discussion

Preoperative differential diagnosis of CE is challenging due to its latency period (asymptomatic period), which may last decades between the initial infection and the onset of clinical symptoms. CE is diagnosed routinely on the basis of clinical symptoms, histopathology, and imaging. Ultrasonography is a widely used technique for abdominal CE and AE lesions [5]. However, when cysts are located in the lungs or other unusual locations, CT or magnetic resonance imaging (MRI) is used [18].

On chest CT, the cyst in the anterior mediastinum of our patient demonstrated features compatible with transitional stages of the WHO-IWGE classification. In this classification, the cyst morphology is visualized using imaging techniques. The detached internal membrane forming flattened pseudo-vesicles corresponds to CE3a (‘water-lily sign’), while the enhancing nodular mass adjacent to the cyst wall suggests a CE3b lesion (Figure 1). According to WHO-IWGE, CE3a and CE3b are transitional stages, distinct from CE1–CE2 (active unilocular or multivesicular cysts) and CE4–CE5 (inactive, degenerative, or calcified cysts). We emphasize that these assignments were derived solely from imaging evidence. Histopathology confirmed echinococcal infection but does not provide staging information.

To support imaging in doubtful CE cases, serology is applied; it is not performed routinely because CE cannot be excluded by negative serology [14]. Diagnosis of mediastinal hydatid cysts is often established intraoperatively during thoracotomy [16], and serological testing is recommended after a hydatid cyst is visualized or for post-treatment monitoring [9]. Microscopic examination of the cyst fluid allows the identification of protoscolices and/or free hooks. Wet-mount, unstained microscopy is often sufficient for diagnosis. Furthermore, the use of various staining methods, such as Ziehl–Neelsen, Wheatley’s trichrome, Ryan’s trichrome blue, Baxby stain, and its modifications, enables better visualization of hooks, regardless of whether they are freely present or within a protoscolex. Cystic material can also be identified in histological tissue sections. In mature echinococcal cysts, an acellular laminated layer and a thin, cellular germinal layer containing hatching vesicles and protoscolices are often observed [19].

Major antigenic compounds of the hydatid fluid can also be immunologically assessed [20]. Specific antibodies can be detected by Western blot and ELISA. Both methods have high sensitivity and are positive in more than 95% of cases. However, antibody production depends on the immune response, affected organ, or the number of hydatid cysts. The sensitivity and specificity of these methods can also vary depending on factors such as the disease stage, co-infections, or underlying health conditions. As a rule, serology and imaging are recommended to be used together when performing screening [21]. In addition, molecular methods, particularly polymerase chain reaction (PCR)-based assays, enable the precise identification of *Echinococcus* DNA in tissue samples [18].

Cystic lesions constitute approximately one-quarter of all mediastinal lesions detected incidentally or during the diagnosis of symptomatic mediastinal abnormalities. Mediastinal echinococcosis is clinically and radiologically indistinguishable from other cystic lesions in the mediastinum [21]. In addition to being a rare disease, extrahepatic echinococcosis typically presents with an atypical clinical presentation, which can mislead clinicians and negatively impact diagnostic and therapeutic decisions, particularly in settings with a low disease incidence. Therefore, delayed diagnosis and inappropriate treatment can negatively impact the course of the disease [22,23].

The course and management of echinococcosis vary depending on the species of tapeworm that causes it [11]. Therefore, it is important to differentiate *E. granulosus* from *E. multilocularis*. Histological criteria combined with serological testing are helpful for this purpose. PCR and a combination of immunohistochemical staining with mAbEm2G11 and mAbEmG3 may also offer diagnostic support if serological testing combined with histopathological examination do not allow for a clear identification of the species. However, in the present case, PCR and additional immunohistochemical staining were not necessary. Histologically, differentiating features are the thickness of the outer layer (CE > 0.15 mm; AE ≤ 0.15 mm), the thickness of the fibrous capsule (CE > 0.6 mm; AE ≤ 0.6 mm), and distinct striation of the outer layer in CE [24] or the presence of necrosis in AE [8,20]. The presence of protoscolices and an outer layer are observed in most cases of CE, but these structures have also been reported in cases of AE [22]. The germinal layer of the cyst produces protoscolices and a brood capsule, which are located within the cyst in a fluid-filled cavity. They should be distinguished from daughter cysts of varying sizes, which can be located both inside and outside the mother cyst [10]. The differential diagnosis also requires consideration of the number of cysts; in this case, it was a single cyst, which is characteristic of CE vs. AE [23].

When the patient was 10 years old, a CE localized in the left lung was diagnosed, and an interlobar cyst was surgically excised. Serological testing was not conducted, but pulmonary CE is often associated with a low rate of seropositivity [9]. An intriguing question is whether the mediastinal CE diagnosed 59 years after pulmonary CE in the same patient represents a relapse of the previously established echinococcosis. Relapses of CE pose a diagnostic and therapeutic challenge for experienced teams of clinicians. Hydatid cysts are known to undergo degeneration, calcification, and structural reorganization, which may allow for internal content displacement, and it is difficult to confirm this phenomenon using serological markers. *E. granulosus*-specific IgG antibodies in blood serum were detected 4 months after the second surgical thoracotomy for the CE hydatid cyst in the anterior mediastinum. These specific IgG antibodies can be detected even when the cyst has been successfully removed [19], thus they cannot be considered indicative of relapse. The serological fluctuations may reflect variability of the assay and therefore should not be interpreted as evidence of relapse.

The imaging in this case provided doubtful results and serology was applied. Although Western blot appears the best test to apply when a one-test approach is chosen, caution is required in differentiating *Echinococcus* species based on the banding pattern. Currently, no evidence-based consensus exists to guide the use of serological tests in the diagnosis of CE [9]. In accordance with the recommendations of the WHO-IWGE, the *Echinococcus species* was identified by specific blot assay ELB to confirm the positive result of a highly sensitive ELISA [14].

Mediastinal hydatid cysts may lead to complications, such as rupture, fistula, embolism, and pressure on vital organs [15]. A high risk of surgical rupture during removal of the interlobar cyst in the left lung could have contributed to the dissemination of cyst content to the anterior mediastinum and its transition into a dormant (inactive) stage. The dynamic nature of hydatid cysts supports the hypothesis of potential displacement and subsequent reactivation [25]. It remains plausible that interlobar CE cysts can migrate within the chest from the lungs to the anterior mediastinum. The emergence of the primary focus of echinococcosis in the anterior mediastinum more than five decades after pulmonary CE seems to be a less likely phenomenon than CE relapse. The growth rate of CE cysts is not well characterized and cyst morphology varies significantly [10]. Cysts typically enlarge at a rate of 1–5 mm per year, but up to 16% of cysts show no growth [24]. This could explain the slow progression of CE over 50 years following excision of an interlobar cyst. The extended growth period of cysts with viable protoscolices has been estimated to be at least 10 months following exposure [26].

The cyst size and growth trajectory of a large-sized mediastinal hydatid cyst measuring 95 mm in diameter more than 50 years after the first surgical intervention for pulmonary CE were consistent with the average cyst diameter of 10.7 cm in patients infected with *E. granulosus* [26].

Based on the available data, the theory of relapse from remote intrathoracic seeding at the time of the first surgery cannot be conclusively confirmed. Alternative scenarios, such as a slow-growing newly acquired CE lesion or a dormant cyst with late reactivation remain possible.

In endemic areas, any cystic lesion in the chest should have a suspicion of *Echinococcus*. However, the patient was probably accidentally infected with *E. granulosus* from a contaminated environment. The source of CE in this case is impossible to establish.

## 5. Conclusions

An *E. granulosus* cyst located in the anterior mediastinum is rare and poses a diagnostic challenge. This case highlights the need to consider echinococcosis in the differential diagnosis of cystic lesions, even decades after primary infection. The combination of histopathological evaluation, high-resolution imaging, and two-step serological testing proved crucial to establish a definitive diagnosis and accurately differentiate the tapeworm species, emphasizing the importance of a multidisciplinary diagnostic strategy to optimize clinical outcomes.

## Data Availability

Data available on request due to ethical reasons. Patient’s records are kept in the archives of the Department of Thoracic Surgery and Transplantation, Pomeranian Medical University, Szczecin, Poland.
